# Numerical Study on the In-Service Welding Stress of X80 Steel Natural Gas Pipeline

**DOI:** 10.3390/ma18030719

**Published:** 2025-02-06

**Authors:** Haiping Tang, Yaping Ding, Guangyou Qiu, Pei Yi, Ziguang Liu

**Affiliations:** 1 Civil Aviation Safety Engineering College, Civil Aviation Flight University of China, Deyang 618307, China; yipei1026064969@163.com (P.Y.); 18790896558@163.com (Z.L.); 2 Department of Transportation and Municipal Engineering, Sichuan College of Architectural Technology, Chengdu 610399, China; 3 Dehong Oil and Gas Branch of Southwest Oil and Gas Pipeline of Pipe China Co., Ltd., Mangshi 678400, China; qiugyou@163.com

**Keywords:** in-service welding, X80 steel pipeline, welding stress, fillet weld, numerical simulation

## Abstract

In this study, the welding stress of in-service welding on the X80 steel pipeline was investigated using the 3D finite element method. The parameters of heat source and axial and hoop welding stress were verified in the experiment. As shown in the results, in the heat-affected-zone (HAZ) location of the pipeline and sleeve, the outer wall was predominantly under compressive stress, while the inner wall was mainly subjected to tensile stress. The hoop stress (*σ_h_*) is greater than the axial stress (*σ_a_*). The maximum hoop stress is primarily concentrated at the connection point between the fillet weld and the sleeve, and its value exceeds the yield strength of X80 steel. Excluding the start–end region, the axial stress distributed in the circumferential direction remains at an almost constant value. The hoop stress values exhibit an approximately symmetric distribution, with relatively higher values at 0° and 180° and relatively lower values at 90° and 270°. Compared with axial stress, the influence of natural gas pressure and flow rate on the hoop stress of the pipeline is more pronounced. When the natural gas pressure increases from 0.5 MPa to 2.5 MPa and the flow rate increases from 1 m/s to 20 m/s, the hoop stress of the pipeline increases by 3.18% and 15.42%, respectively. Moreover, the influence of the preheating temperature on the axial stress of the sleeve is highly prominent. When the preheating temperature is elevated from 20 °C to 300 °C, the axial welding stress of the sleeve increases by 115.3%. These results indicate that maintaining the natural gas pressure at 1 MPa, keeping the flow rate below 12 m/s, and controlling the preheating temperature at approximately 50 °C can enhance the performance of the fillet weld during the in-service welding of X80 steel pipelines.

## 1. Introduction

Long-distance pipelines play an important role in the oil and gas industry. However, the strength of the pipeline is affected by geological disasters, corrosion, and other factors during the service process [1,2]. It will cause significant economic losses and environmental pollution once the pipeline fails. In order to ensure the strength of a defected pipeline, type-B sleeves are widely used to reinforce defected pipelines [3,4].

**Figure 1:**
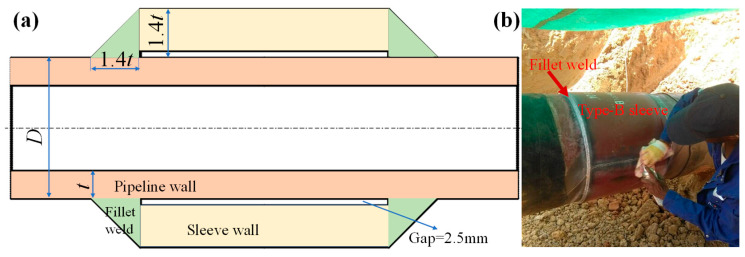
The in-service welding: (**a**) a schematic diagram of sectional structure; (**b**) in-service welding engineering.

The structure of type-B sleeves is depicted in Figure 1a, and type-B sleeves are connected to the pipeline through a fillet weld, forming a closed gap space to prevent oil and gas leakage. It can minimize unfavorable effects for pipeline users and operators. Figure 1b shows the in-service welding on the pipeline, and the pipeline remains in operation. Burn-through and cracking in the heat-affected zone of the fillet weld are the two fundamental problems for in-service welding on pipelines [5]. However, with the increase in pipeline steel grade, the wall thickness and diameter of long-distance pipelines also increase accordingly [6]. Up to now, X80 pipeline steel is the highest-strength steel that is widely used in long-distance natural gas pipelines, such as the West–East Gas Pipeline Project [7]. The burn-through can be neglected for the X80 steel pipeline due to the wall thickness being more than 6.4 mm [8]. In contrast, welding stress will become more complex for increasing the wall thickness of pipelines, and a greater risk must be faced [9,10,11,12].

In order to improve the quality and reduce the stress of in-service welding on the X80 steel pipeline, Zhang et al. found that the welding stress distribution became more uniform as the fillet weld size increased, and the fillet weld size was suggested to be larger than 1.4 ***t*** [13]. Asl et al. investigated the burn-through at the in-service welding of 316 stainless-steel pipelines using the thermo-mechanical numerical method. The results showed that there is higher stress for the positions of 180° and 360°, and the thermal concentration on the pipe wall can be reduced by arranging the shape and location of the bead [14]. Alian et al. established a 3D thermo-mechanical model to study the stress of an 8″, X65 pipe with a single-pass weld. They proposed that the back-step welding scheme induced the lowest average residual stresses compared to other welding schemes [15]. Wu et al. conducted research on burn-through during the in-service welding of pipelines with corrosion defects. They found that the maximum radial deformation, von Mises stress, and hoop stress in the defect area all increase when the medium pressure rises. Moreover, the depth of the defect has a pronounced influence on the radial deformation and the stresses [8,16]. Zhang et al. analyzed the local heat treatment to adjust the residual stress of the repaired welded joint of the in-service pipeline using the finite element method, and they revealed that the local heat treatment is very useful for reducing residual stress [17]. Milenin et al. presented a novel criterion of the plastic instability of a corroded X65 pipeline under multi-pass in-service weld deposition repair and determined the allowable heat input parameters and optimal deposition scheme from the point of view of possible burn-through and excessive residual deformation of the pipe [18]. Guo et al. investigated the influence of the welding heat input of a B-type sleeve circumferential fillet weld on the temperature and stress field at the circumferential pipe weld. The results displayed that the axial stress peak of the inner wall of the pipe girth weld reduces with the increase in welding heat input of sleeve circumferential fillet weld, and the axial deformation peak of the inner wall of the pipe girth weld increases first and then decreases with the increase in the welding heat input of the sleeve circumferential fillet weld [19]. In addition to the research reports on the stress of in-service welding mentioned above, the temperature of in-service welding is of interest [20,21,22,23]. However, to further examine the stress of in-service welding, the effects of flowing media inside high-grade pipelines still remain to be investigated.

In this work, numerical simulation and an experiment were carried out to investigate the welding stress of in-service welding on the X80 steel natural gas pipeline. A 3D finite element model was established according to the experimental structure of an X80 steel pipeline. The effects of the natural gas pressure, flow rate, and preheat temperature on the welding stress were discussed based on the validated numerical model.

## 2. Experiment and Materials

### 2.1. Experiment Procedure

**Figure 2:**
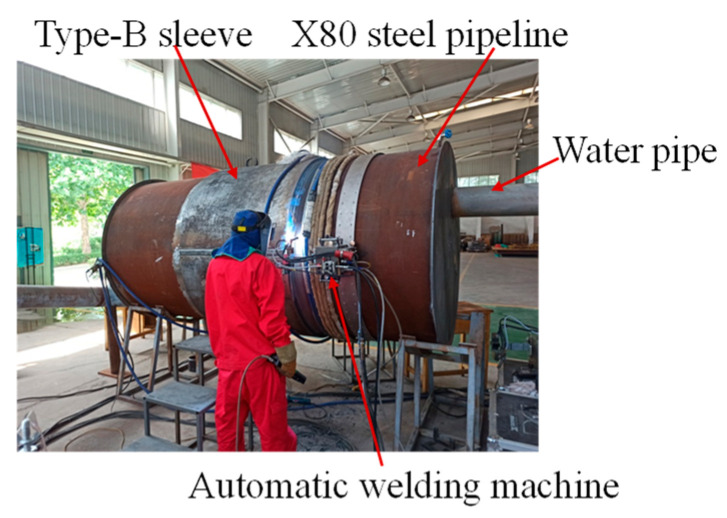
The experimental device.

**Figure 3:**
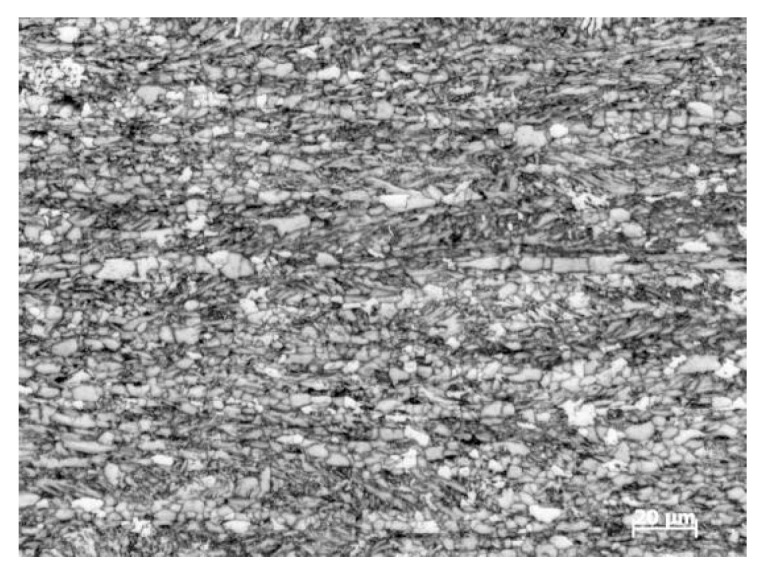
Microstructure of X80 steel.

Considering safety, the natural gas was replaced with water in the pipeline. The in-service welding experiment was established in the National Pipeline Network Group Research Institute. As shown in Figure 2, the experimental device consisted of test pipeline, circulating water pipeline, and water pump. The test pipeline is X80 pipeline steel, with an external diameter of 813 mm and wall thickness of 12.7 mm. The microstructure of X80 is shown in Figure 3; it is primarily composed of quasi-polygonal ferrite, acicular ferrite, granular bainite, and black M-A constituents, presenting a banded distribution pattern. The type-B sleeve is also X80 pipeline steel. The thermal physical parameters of X80 pipeline steel and water are listed in Table 1 and Table 2, respectively.

**Figure 4:**
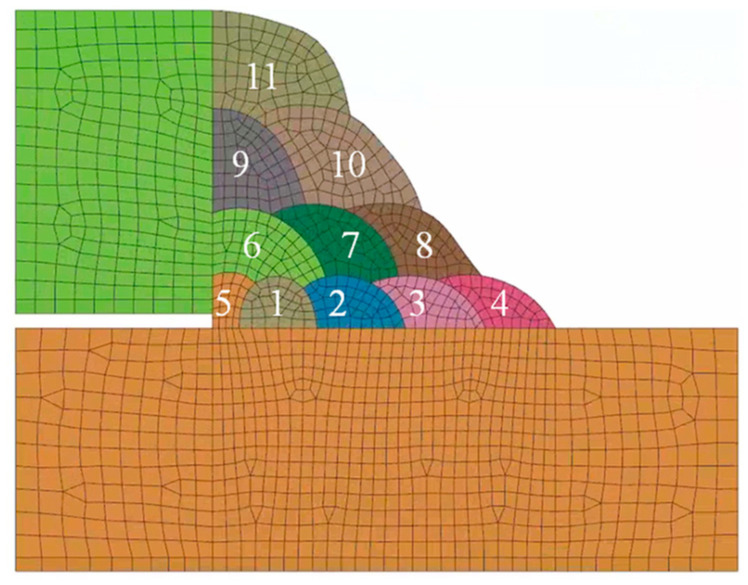
The structure of fillet weld and welding sequence.

The welding material E91T1-GM with a diameter of 1.2 mm is deposited in the filling passes, and E8018-C3 with a diameter of 3.2 mm is deposited in the root passes. The fillet welds consist of four layers and 11 passes, with the numbers from 1 to 11 representing the welding sequence of fillet weld, and the deposition sequences are presented in Figure 4. The No.1 to No.4 passes are the root passes, which are welded via shield metal arc welding (SMAW). However, No.5 to No.11 are the filling passes, which are welded via automatic gas metal arc welding (GMAW) with 80% Ar + 20% CO_2_ shielded gas. The weld formation is accomplished in sequence from 1 to 11. Two kinds of welding materials were selected for the fillet weld. The welding parameters are recorded in Table 3.

### 2.2. Test of the Welding Stress

**Figure 5:**
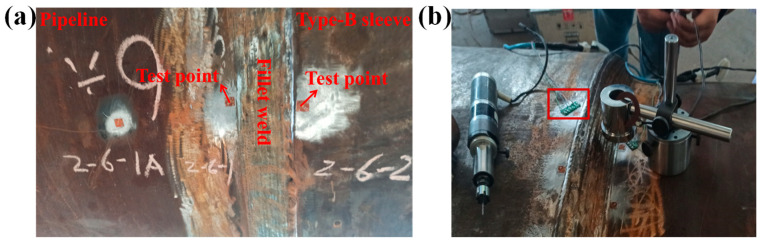
The welding stress test: (**a**) the test location; (**b**) stress test.

To obtain the welding stress, the hole-drilling test was carried out according to ASTM E837 standard [24]. As shown in Figure 5a, the stresses on the pipeline and type-B sleeve near the fillet weld were measured using the hole-drilling method. The locations of 0°, 30°, and 300° were selected as the test points. Strain gauge system is HK21 (Shandong Huawin Electrical and Mechanical Technology Co., Ltd., Jinan, China), which consists of data acquisition system, control device, and blind hole device. Strain gauge is BX120-2CA (Beijing Yiyang Yingzhen Testing Technology Co., Ltd., Beijing, China) rosettes. Each rosette gauge consists of three strain gauges, two of which are perpendicular to each other, and the third is at an angle of 45° or 135° to any of the first two, as illustrated in Figure 5b.

The principal stresses, $\sigma_{1}$ and $\sigma_{2}$, and the principal stress direction angle, θ, were calculated according to Equations (1)–(3) based on the measured strain values $\varepsilon_{1}$, $\varepsilon_{2}$ and $\varepsilon_{3}$.

(1) $$\sigma_{1}=\frac{E}{4A}\left(\varepsilon_{1}+\varepsilon_{3}\right)-\frac{E}{4B}\sqrt{{\left(\varepsilon_{1}-\varepsilon_{3}\right)}^{2}+{\left(2\varepsilon_{2}-\varepsilon_{1}-\varepsilon_{3}\right)}^{2}}$$

(2) $$\sigma_{2}=\frac{E}{4A}\left(\varepsilon_{1}+\varepsilon_{3}\right)+\frac{E}{4B}\sqrt{{\left(\varepsilon_{1}-\varepsilon_{3}\right)}^{2}+{\left(2\varepsilon_{2}-\varepsilon_{1}-\varepsilon_{3}\right)}^{2}}$$

(3) $$tg2\theta =\frac{2\varepsilon_{2}-\varepsilon_{1}-\varepsilon_{3}}{\varepsilon_{3}-\varepsilon_{1}}$$

where *A* and *B* are stress release factors obtained from calibration according to China shipping industry-standard CB/T 3395 [25]. The hoop and axial stress values in the pipe welded joint can be further calculated by:

(4) $$\sigma_{hoop}=\sigma_{1}\cos^{2}\theta +\sigma_{2}\sin^{2}\theta$$

(5) $$\sigma_{axial}=\sigma_{1}\sin^{2}\theta +\sigma_{2}\cos^{2}\theta$$

## 3. Finite Element Modeling

### 3.1. Geometry and Meshing

**Figure 6:**
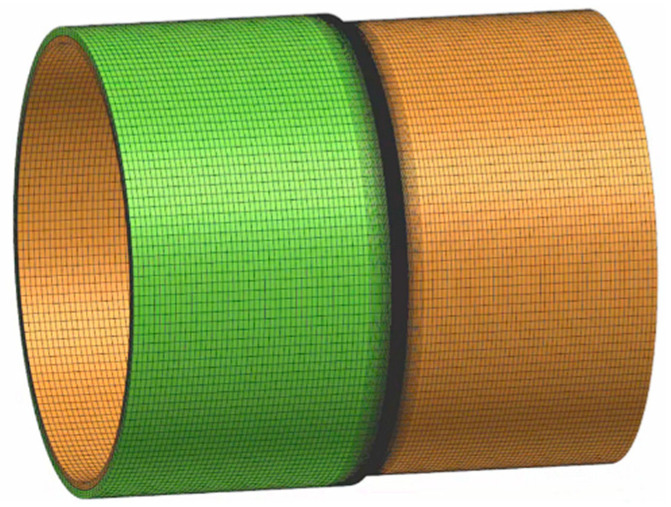
The finite element model of in-service welding on X80 steel pipeline.

To investigate the in-service welding on X80 streel pipeline and reduce computation time, the longitudinal butt welds of the type-B sleeve are not considered in this study. As depicted in Figure 6, the in-service welding model of X80 steel pipeline was established in the 2019-version of SYSWELD software. The numerical model was built strictly in accordance with the actual testing pipeline, fillet weld, and type-B sleeve. In this model, the pipeline length and sleeve length are 2 m and 1 m, respectively. The wall thickness and outer diameter of pipeline are 12.7 mm and 813 mm, respectively. Since the fillet weld of the type-B sleeve is closed girth weld, and the stress is triaxial stress, a 3D finite element model has higher calculation accuracy than the 2D model. Meanwhile, the mesh of weld and HAZ, where temperature and stress change dramatically during welding, was refined, and its adjacent area was divided by the coarse mesh to balance the contradiction between calculation accuracy and efficiency.

### 3.2. Boundary Conditions

The fillet welds at both sides of the type-B sleeve were welded using the same processes simultaneously; the symmetry boundary was used in the numerical simulation to reduce the size of large-size 3D model and improve efficiency. Fixed restrains were applied at both ends of the pipeline to confirm that the model was restrained as the actual pipeline and guarantee the calculation accuracy of the welding stress.

### 3.3. Thermal and Mechanical Numerical Analysis

The double ellipsoidal heat source model (DEHSM) proposed by Goldak was employed to simulate the in-service welding process [23]. The volumetric heat flux of the heat source model is described as follows:

(6) $$q_{i}=\frac{6\sqrt{3}f_{i}Q}{\pi^{3/2}a_{i}bc}\exp\left\{-3\left[{\left(\frac{x-vt}{a_{i}}\right)}^{2}+{\left(\frac{y}{b}\right)}^{2}+{\left(\frac{z}{c}\right)}^{2}\right]\right\}\;i=f,r$$

where *f* and *r* denote the front and rear semi-ellipsoid at an arbitrary point (*x*, *y*, *z*); *x*, *y*, *z* are the Cartesian coordinates; *q_f_* and *q_r_* are the front-half and rear-half volumetric heat flux of the DEHS model, respectively; *a*, *b*, *c* describe the dimensions of the DEHSM; *f_f_* and *f_r_* are the energy coefficients corresponding to the front and the rear of ellipsoid heat source, respectively; $f_{f}+f_{r}=2$, *v* is the welding speed; *t* is the time. The heat input *Q* is calculated as:

(7) Q=ηUI

where *U* and *I* are the voltage and current of in-service welding, respectively. *η* denotes the welding heat efficiency.

The coefficient of natural convection is about 25 W/(m^2^·K) at 20 °C, and the radiation’s coefficient is calculated by the formula recommended by SYSWELD software. The total heat transfer coefficient of the outside surface *h_o_* is equal to the sum of convection and radiation coefficient and can be expressed as follows:

(8) $$h_{o}=0.8\times 5.67\times 10^{-8}\left(T^{4}-T_{0}^{4}\right)+25$$

where *T* and *T*_0_ represent the temperature of the pipeline surface and ambient temperature, respectively.

The heat transfer mechanism between the inside surface of pipeline and the flowing gas is presumed to be forced convection, and the gas is methane. Some thermal–physical properties of methane are recorded in Table 4, and the heat transfer coefficient is given as:

(9) $$h_{i}=\frac{Nu\lambda }{D}$$

where *h_i_* is the heat transfer coefficient of pipeline inside surface, *D* is the outside diameter of the pipe, and λ is the thermal conductivity. *Nu* and *Pr* are Nusselt Number and Prandtl number, respectively, which can be calculated as follows [22]:

(10) Nu=0.023Re0.8Pr0.4

(11) $$\mathrm{Pr}=\frac{\mu_{f}C}{\lambda }$$

(12) Re=ρwDμf

where *C* represents the constant-pressure specific heat, *μ_f_* denotes the coefficient of dynamic viscosity, *ρ* and *w* are the density and velocity of the gas, respectively.

The thermal result of each node was extracted for mechanical analysis. The thermal-dependent mechanical parameters were assigned to the mechanical model. In the subsequent mechanical analysis, the transient and residual stress distributions are calculated by taking the previously computed temperature field as a load. The geometry model establishment, meshing process, and activation of weld elements are consistent with those in the thermal analysis. The elastic behavior of the material is simulated in accordance with the isotropic Hooke’s law. The impact of temperature on the mechanical behavior is accounted for via the coefficient of thermal expansion. As for the plastic behavior, a rate-independent plasticity model is utilized. The total strain can be decomposed and expressed as below:

(13) $$\varepsilon_{total}=\varepsilon_{e}+\varepsilon_{p}+\varepsilon_{th}$$

where *ε_e_* is elastic strain, *ε_p_* is the plastic strain, *ε_p_* is the thermal strain.

The isotropic strain-hardening model was employed to describe the initial yield and cyclic yield behavior. During mechanical simulation, the evolution of the yield surface radius was described as:

(14) $$\sigma =\sigma_{0}+Q_{\inf}\left(1-e^{-b{\bar{\varepsilon }}^{pl}}\right)$$

where $\sigma_{0}$ is the yield stress of zero plastic strain, $Q_{\inf}$ and *b* are material parameters with values of 180 MPa and 10, and ${\bar{\varepsilon }}^{pl}$ is the equivalent plastic strain.

## 4. Results and Discussion

### 4.1. Temperature and Microstructure Analysis

**Figure 7:**
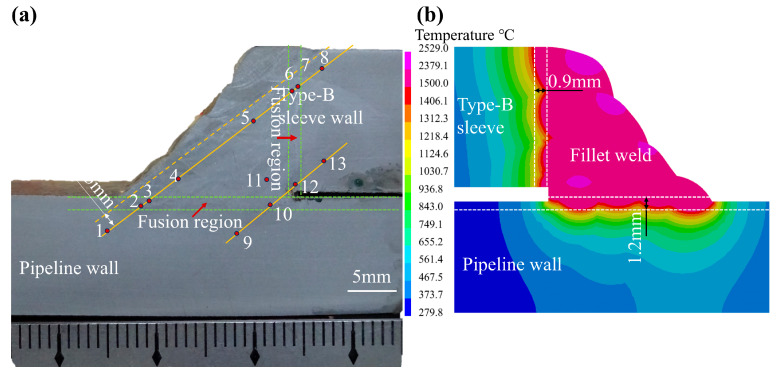
Weld morphology of the test pipeline and numerical simulation: (**a**) the experiment; (**b**) the simulation.

The structure and temperature field of the fillet weld are shown in Figure 7. It can be observed that the simulations of the fusion zone exhibit a good agreement with the experimental results. When comparing the weld penetration depth between numerical simulation and experiment, we find that the weld penetration depth of the pipeline is about 1.2 mm, and the weld penetration depth of the type-B sleeve is about 0.9 mm. This indicates that the proposed parameters of the heat source function are suitable for the welding process, and the heat source parameters are listed in Table 5. The numbers ranging from 1 to 13 denote the positions for hardness testing of the fillet welds and their corresponding heat-affected zones. 

The hardness of the reference point in Figure 7 was measured using a Vickers hardness tester (Suzhou Nanguang Electronic Technology Co., Ltd., Suzhou, China) with 10 kgf (Hv_10_) and a dwell time of 10 s. The hardness test results are presented in Table 6. Evidently, the hardness of the fillet weld spans from 194 (HV_10_) to 232 (HV_10_). Concurrently, the hardness of the HAZ ranges from 203 (HV_10_) to 245 (HV_10_). The region of the parent material adjacent to the HAZ exhibits a hardness range of 210 (HV_10_) to 225 (HV_10_). The excessive hardness can enhance the crack sensitivity, whereas insufficient hardness will lead to a reduction in strength. When comparing the hardness of the fillet weld and HAZ, it is determined that the Vickers hardness of the fillet weld complies with the requirements specified in the GB/T 31032-2023 standard [26].

**Figure 8:**
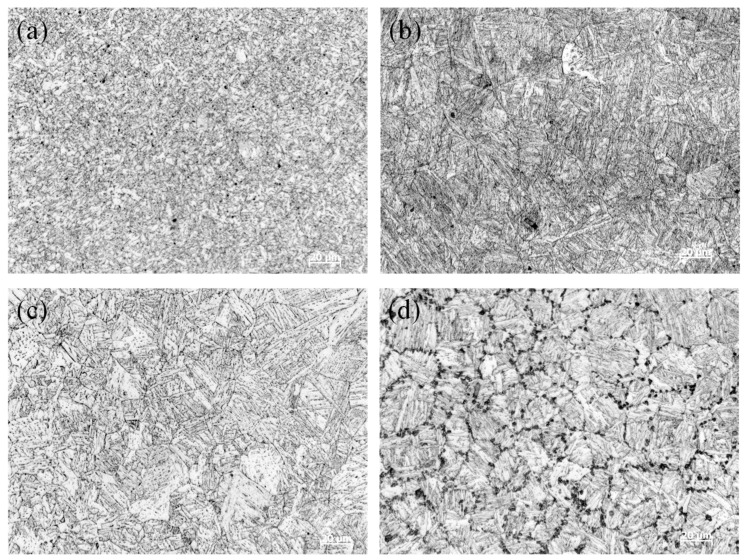
The microstructure (**a**) welding pass, (**b**) coarse-grain zone, (**c**) fine-grain region, (**d**) sub-critical coarse-grain zone.

The microstructure of the fillet weld is presented in Figure 8. In Figure 8a, the microstructure of the weld is mainly composed of needle-shaped ferrite, with the columnar crystal interfaces being faintly distinguishable. As demonstrated in Figure 8b, the coarse-grain zone primarily consists of granular and lath bainite. The only differences are manifested in the content and dimensions of each phase. Figure 8c reveals that the microstructure of the fine-grain zone is fine, which consists of quasi-polygonal ferrite, lath bainite, and granular bainite. Regarding the sub-critical zone of the HAZ, its microstructure is depicted in Figure 8d. The sub-critical zone displays a relatively coarse microstructure, characterized by distinct austenite grain boundaries and chain-like M-A constituents distributed along these boundaries.

This phenomenon can be mainly attributed to the fact that when the temperature ranges between Ac1 and Ac3, the lath martensite and the mixed structure predominantly composed of lath bainite within the coarse-grain structure are not fully austenitized. During rapid heating and cooling, the non-austenitized original mixed structure precipitates more M-A components. Meanwhile, the austenitized structure undergoes recrystallization during the rapid cooling process, resulting in a relatively fine chain-like structure that contains lath bainite and is distributed along the grain boundary. Consequently, a substantial number of chain-like M-A components distributed along the grain boundaries are observed in the sub-critical region.

### 4.2. Welding Stress Field Analysis

**Figure 9:**
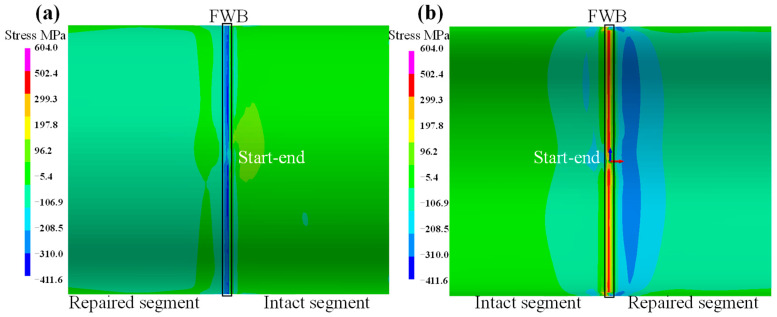
Axial stress on pipeline: (**a**) external surface; (**b**) internal surface.

The axial stress on the pipeline is shown in Figure 9. It is evident that the axial stress at the fillet weld location exhibits a significant difference between the external and internal surfaces. Additionally, the axial stress on the repaired side manifests as compressive stress, while that on the intact side is tensile stress. The magnitude of the stress on both external and internal surfaces is approximately 96 MPa. As depicted in Figure 9a, the axial stress distribution along the pipeline is presented on the external surface of the pipeline. At the fillet weld location, the axial stress is in a compressive state, with the corresponding peak value of axial stress reaching approximately −320 MPa. In the vicinity of the fillet weld zone, the axial stress remains compressive, yet its peak value drops to around −100 MPa. Notably, the axial compressive stress at the weld start point is lower than that at other fillet weld locations, with a value of −200 MPa.

Figure 9b illustrates the axial stress on the inner surface of the pipeline. Evidently, a significant tensile stress is distributed along the fillet weld location, with a peak value of 400 MPa. Nevertheless, the tensile stress at the start–end location reduces to 96 MPa. On the repaired segment side of the pipeline, there exists a prominent compressive stress zone, and its peak value reaches −300 MPa. However, on the both right and left sides of the fillet weld location, the axial stress is compressive; on the intact segment side far away from the fillet weld, the stress transforms into tensile stress.

**Figure 10:**
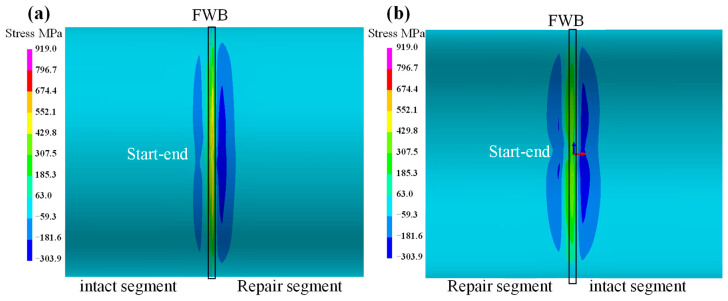
The hoop stress on pipeline: (**a**) external surface; (**b**) internal surface.

Figure 10 shows the hoop stress distribution on the external and internal surface of the pipeline. It is evident that the distribution patterns of hoop stress on the external and internal surfaces are identical. At the fillet weld location, the hoop stress is tensile stress. Notably, the tensile stress value gradually decreases as the distance from the start–end position increases. Conversely, the left and right fillet welds face compressive stress. In the area far from the fillet weld, the stress value is approximately −20 MPa, and it is uniformly distributed. Moreover, on the intact segments of both the external and internal surfaces, there exists a distinct compressive stress area, with the stress value reaching 303 MPa. Figure 10a shows the distribution of hoop stress on the external surface of the pipeline. At the fillet weld location, a significant tensile hoop stress is apparent. The peak value, exceeding 570 MPa, is located at the start–end position. In contrast, the hoop stress on the internal surface of the pipeline exceeds 350 MPa, which is presented in Figure 10b.

Comparing the stress on the outer surface with that on the inner surface, it can be found that the hoop stress at the fillet weld location is greater than the axial stress. Guo et al. reported that high hoop stress is the reason for the transverse crack in the fillet weld [27].

**Figure 11:**
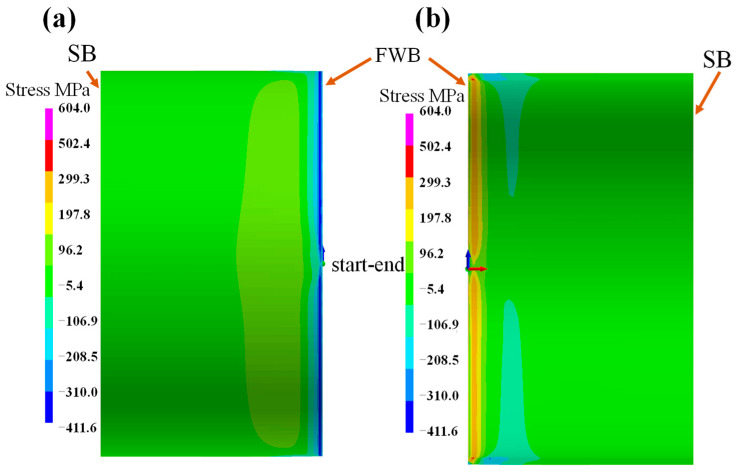
The axial stress on sleeve: (**a**) external surface; (**b**) internal surface.

The axial stress on the external and internal surface of the sleeve is depicted in Figure 11. With the exception of the region adjacent to the fillet weld, the stress distribution patterns and values on both the inner and outer surface of the pipeline are essentially identical. As shown in Figure 11a, on the external surface of the sleeve, the axial stress in the close vicinity of the fillet weld boundary (FB) is in a compressive stress state. The peak value exceeds −310 MPa, while it decreases to −106 MPa at the start–end location. As the distance from the FB progressively increases, the axial stress initially ascends gradually to approximate 190 MPa and subsequently declines to −5.4 MPa. Figure 11b illustrates the axial stress distribution on the internal surface of the sleeve. It can be found that the tensile stress in close proximity to the FB is highly conspicuous. With the exception of the starting point position, where the stress value is 205 MPa, the stress is uniformly distributed along the circumferential direction, with a magnitude of 305 MPa. Moreover, in the region adjacent to the symmetrical boundary (SB), the axial stress distribution exhibits relatively high uniformity, with a value of approximately −5.4 MPa. Therefore, the axial stress on the internal surface of sleeve is more harmful.

**Figure 12:**
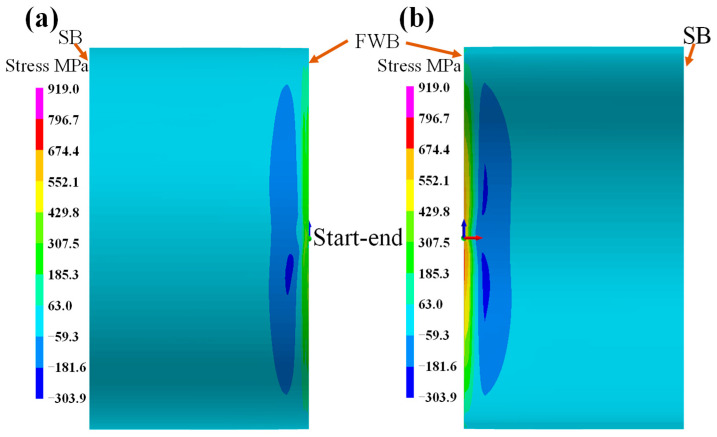
The hoop stress on sleeve: (**a**) external surface; (**b**) internal surface.

Figure 12 shows the hoop stress on the external and internal surfaces of the sleeve. It can be observed that the hoop stress distribution on the external surface is the same as the internal surface. Close to the FB, tensile stress is predominant, and the peak tensile stress is concentrated at the start–end location. Near the FB for both the external and internal surfaces, there is a notable compressive stress area, with a peak value of −285 MPa. Regarding the location from the FB to the symmetric boundary, the hoop stress decreases to approximately −5 MPa. As seen in Figure 12a, the peak hoop tensile stress on the external surface is more than 430 MPa. Meanwhile, as depicted in Figure 12b, the peak hoop tensile stress on the internal wall exceeds 674 MPa.

By comparing the hoop and axial stresses on both the external and internal surfaces of the sleeve, it can be revealed that the stress magnitude on the internal surface is greater than that on the external surface. Moreover, the hoop stress is significantly higher than the axial stress. In particular, the hoop tensile stress exceeds the yield stress of X80-grade pipeline steel, which poses a risk for crack growth [28].

**Figure 13:**
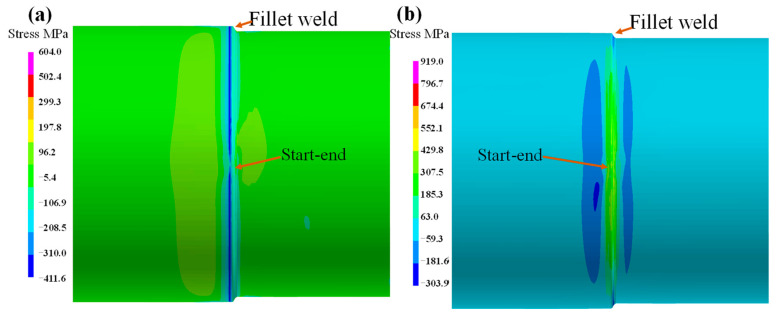
The welding stress on fillet weld: (**a**) axial stress; (**b**) hoop stress.

Figure 13 shows the welding stress distribution on the external surface of the fillet weld. It can be found that the axial stress of the fillet weld is different from that on the sleeve and pipeline. However, the hoop stress on the weld seam transitions from a continuous distribution between the pipeline and the casing. As shown in Figure 13a, the axial stress along the fillet weld is in a range of −106 to −208 MPa; however, the axial stress on the weld starting location decreases within a range of −5.4 MPa to 96.2 MPa. The axial stress on the sleeve adjacent to the fillet weld is significant compressive stress, with a value exceeding −310 MPa. Meanwhile, the axial stress on the pipeline near the fillet weld is tensile stress, with a value of 96 MPa. Figure 13b demonstrates the hoop stress distribution on the surface of the fillet weld. As the position approaches the starting point, the tensile stress become increasingly prominent, with values ranging from −107 MPa to 197 MPa. Thus, the effect of the hoop stress on the safety of the fillet is more significant than axial stress.

**Figure 14:**
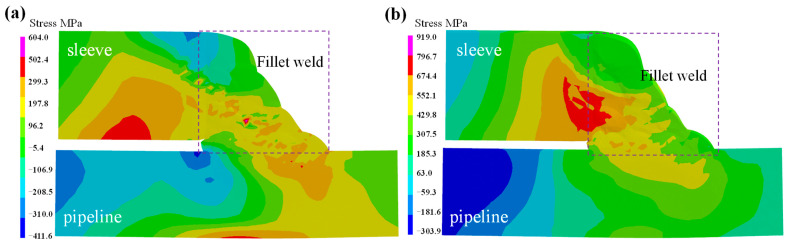
The stress distribution on the fillet weld cross-section: (**a**) axial stress; (**b**) hoop stress.

Figure 14 displays the maximum welding stress on the cross-section of the fillet weld. It can be observed that both the axial and hoop stresses on the left of the fillet weld are in a compressive state, whereas the stresses on the right of the fillet weld are in a tensile state. Figure 14a shows the axial stress distribution. Evidently, on the internal surface of the sleeve, a tensile stress concentration zone with a value over 400 MPa is present. Meanwhile, at the fillet weld, a high axial tensile stress zone is positioned in the No.7 pass, and its value also exceeds 400 MPa. Figure 14b shows the maximum hoop stress on the cross-section of the fillet weld. It can be clearly seen that the peak tensile stress on the pipeline exceeds 674 MPa and is located in the middle layer, which serves as the connection part between the fillet weld and the sleeve. Apart from this specific location, all other areas of the fillet weld face tensile stress. Moreover, the area with stress exceeding 429 MPa occupies the majority of the entire cross-section of the weld seam. Therefore, many fillet weld failures are caused by cracks, while the high stress within the fillet weld is the main reason [29].

Based on the above-mentioned analysis, the hoop stress plays a dominant role in the in-service welding of the X80 steel pipeline. Notably, the hoop stress in the fillet weld is greater than that in the sleeve and pipeline. Its value surpasses the yield strength of X80 steel, thereby increasing the risk of cracking.

**Figure 15:**
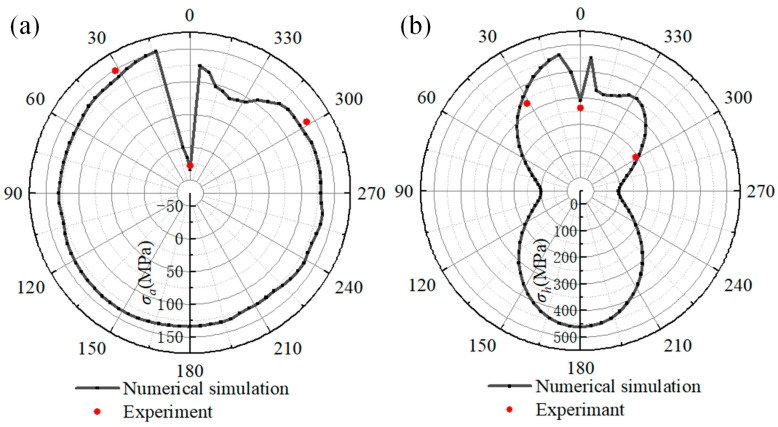
The stress distribution along the reference path of numerical simulation and experiment: (**a**) axial stress; (**b**) hoop stress.

To verify the welding stress obtained from the numerical simulation, the reference paths along the welding direction on the pipeline and sleeve were selected to analyze the welding stress. The reference path traverses the position of the stress-testing point on the pipeline and sleeve. The stress along the reference path on the pipeline is shown in Figure 15. Figure 15a illustrates the axial stress of the reference path. The axial stress displays complex variations from the 315° to 15° location. When comparing the numerical results with the experimental data, it can be observed that the experimental results for the locations of 0°, 30°, and 300° are −28.1 MPa, 148.9 MPa, and 138.3 MPa, respectively. The numerical results are −34.2 MPa, 138.4 MPa, and 126.5 MPa at the corresponding positions on the pipeline, respectively. As Figure 15b shows, the hoop stress values obtained from numerical simulation are 289.6 MPa, 361.9 MPa, and 208.5 MPa at the 0°, 30°, and 300° locations. In contrast, the hoop stress values of the experimental results at the corresponding positions are 262.2 MPa, 335.2 MPa, and 192.4 MPa, respectively.

**Figure 16:**
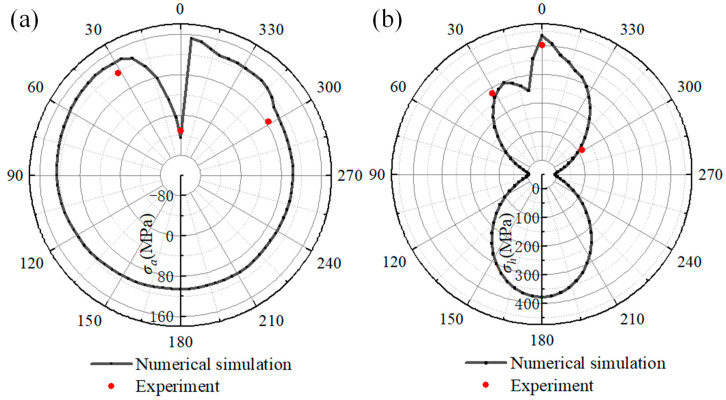
The welding stress on reference path of the sleeve: (**a**) axial stress; (**b**) hoop stress.

The welding stress on the sleeve reference path is presented in Figure 16. As shown in Figure 16a, the simulated axial stress distribution along the reference path is relatively uniform, except within a range of 345° to 30°. At the start–end location, the axial stress is −45 MPa, representing an increment of −14 MPa compared to the experimental result. At the 30° position of the reference path, the simulated axial stress reaches a peak value of 140 MPa, while the experimental result is 118.3 MPa. As the position progresses to 300°, the simulation and experiment results decline to 106.5 MPa and 84.4 MPa, respectively.

Figure 16b shows the hoop stress distribution along the reference path. It can be found that the welding stress at the 90° and 270° positions is −4 MPa, which is lower than that at other locations. When comparing hoop stress distributions, it can be observed that the maximum hoop stress in the numerical simulation reaches 434 MPa and is located at 0°, which corresponds to the start–end location of the fillet weld. As the position progresses from 0° to 30°, the simulated hoop stress first decreases from 434 MPa to 245 MPa and then increases to 266 MPa. The experimental hoop stress values at the 0° and 30° locations also decline from 402.2 MPa to 282.3 MPa. Additionally, at the 300° location, the hoop stress values for numerical simulation and experiment are 96.4 MPa and 114.2 MPa, respectively.

Based on the welding stress data presented in Figure 15 and Figure 16, the principal factors contributing to the phenomenon of the welding stress distribution along the path are as follows. Owing to the closed-loop fillet weld structure, the start–end zone undergoes two thermal cycles during each welding pass. This process specifically induces the generation of compressive axial stress within the start–end zone. Outside the start–end area, the axial stress along the reference path remains relatively consistent. In contrast, the distribution of hoop stress along the reference path adheres to a distinct pattern. It is characterized by relatively higher magnitudes at the 0° and 180° positions and lower values at the 90° and 270° positions. As depicted in Figure 7, the welding heat input during passes No.1 through No.4 is primarily applied to the outer wall of the pipeline. Conversely, the heat input for the subsequent fillet passes is predominantly concentrated on both sides of the sleeve. What’s more, the welding heat input effect on the sleeve wall thickness is uneven during the deposition of the fillet weld. Significantly, the circumferential weld at the end of the casing generates stress that is difficult to dissipate. This situation results in the stress level on the casing being higher than the residual stress present on the pipeline.

By comparing the simulated and experimental results, it can be seen that the minimum and maximum errors of the simulated and tested values are 7.96% and 21.7%, respectively. The presence of a certain deviation can be attributed to several factors. Firstly, in the numerical modeling, the actual material processing before welding was not considered. Secondly, there are differences in material properties between the numerical simulation and the real material. Thirdly, there is a position error between the experiment and the simulation. Additionally, the longitudinal welds also affect the welding stress of fillet welds, yet, in this study, the longitudinal welds were neglected. However, the variation law of welding stress along the reference path is in good agreement with the experimental results. The comparison results validate the reliability of the welding stress results [30,31].

### 4.3. Effect of Gas Pressure on the Welding Stress

**Figure 17:**
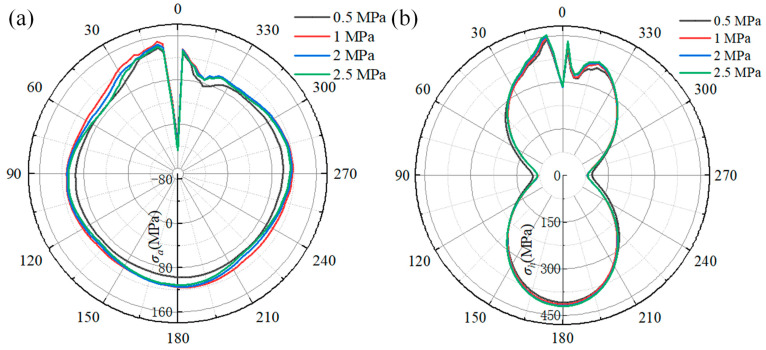
The effect of gas pressure on the welding stress of the pipeline: (**a**) the axial stress curves; (**b**) the hoop stress curves.

The effect of gas pressure on the welding stress of the pipeline is depicted in Figure 17. Figure 17a shows the welding axial stress distribution along the reference path. The distribution of curves shows that the maximum welding axial stress is located at 9° of the pipeline. As the natural gas pressure increases from 0.5 MPa to 2.5 MPa, the maximum welding axial stress first increases from 140 MPa to 151 MPa and then decreases to 142 MPa. Comparing the welding axial stress at 90°, 180°, and 270°, it can be found that the welding axial stress on these locations is unaffected by the natural gas pressure, except at a natural gas pressure of 0.5 MPa.

Figure 17b shows the relationship between the hoop stress distribution and natural gas pressure. It can be found that the maximum welding hoop stress along the distribution curve is at 9°. As the natural gas increases from 0.5 MPa to 2.5 MPa, the welding hoop stress at 9° increases from 440 MPa to 453 MPa, and the welding hoop stress at 180° increases from 408 MPa to 420 MPa, while the welding hoop stress at 0° (start–end location of fillet weld) is hardly affected by the natural gas pressure. Comparing the welding hoop stress at 90° and 270°, the welding hoop stress at 0.5 MPa is different from that at other pressures, and the welding hoop stress is almost uniform when the pressure is more than 0.5 MPa.

The axial stress and hoop stress induced by the pressure can be calculated using Equations (15) and (16)**.** It can be found that axial stress and hoop stress induced by the pressure are much smaller than the welding stress. The natural gas pressure causes the stress to be in a tensile stress state, which is equivalent to applying prestress to the pipeline. Meanwhile, the natural gas pressure is proportional to the heat exchange coefficient, which accelerates the cooling rate and exacerbates the welding stress.

(15) $$\sigma_{ap}=\frac{PD}{4t}$$

(16) $$\sigma_{\theta p}=\frac{PD}{2t}$$

**Figure 18:**
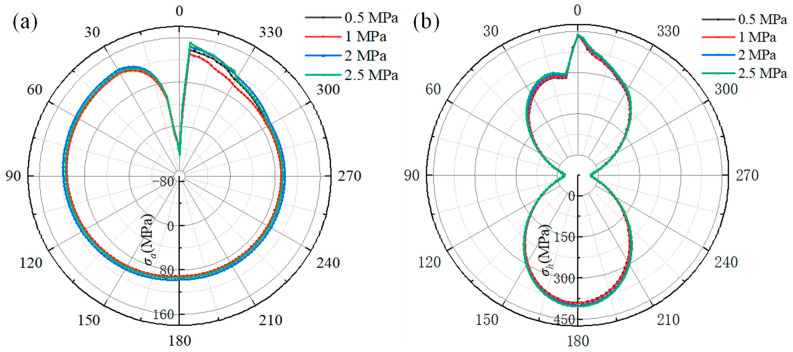
The effect of gas pressure on the welding stress of the sleeve: (**a**) the axial stress; (**b**) the hoop stress.

The relationship between welding stress on the sleeve and natural gas is shown in Figure 18. Figure 18a shows the effect of the natural gas on the welding axial stress on the sleeve. It can be seen that the axial stress curves coincide for pressures of 0.5 MPa and 1.5 MPa, and the distribution of the axial stress curves is also the same for pressures of 2 MPa and 2.5 MPa. The axial stress increases with the increasing natural gas pressure. As the natural gas pressure increases from 0.5 MPa to 2.5 MPa, the axial stress at the location of 351° increases from 147 MPa to 163 MPa, while the axial compressive stress at 0° increases from 40 MPa to 51 MPa.

Figure 18b shows the effect of natural gas pressure on the hoop stress of the sleeve. It is obvious that the hoop stress at the locations of 90° and 180° is almost unaffected by the natural gas pressure, while the hoop stress at 15° is most influenced by the increasing natural gas pressure. As the natural gas pressure increases from 0.5 MPa to 2.5 MPa, the hoop stress at 0° location grows from 432 MPa to 437 MPa, the hoop stress at 180° location increases from 390 MPa to 403 MPa, and the hoop stress at 15° location increases from 291 MPa to 309 MPa, an increase of 15 MPa.

Based on the welding stress distribution along the reference path from Figure 17 and Figure 18, when the gas pressure increases from 0.5 MPa to 2.5 MPa, the peak axial and hoop stresses of the pipeline increase by 1.9% and 3.18%, respectively, and the peak axial and hoop stresses of the sleeve increase by 2% and 1.17%, respectively. Notably, the increase in natural gas pressure does not cause a continuous increase in axial stress on the pipelines and sleeve. Specifically, when the pressure exceeds 1 MPa, the axial stress gradually increases. It can be revealed that the welding stress on the pipeline is more significantly affected compared to that on the sleeve with the increase in gas pressure.

As reported in [20], with an increase in pressure, the density of natural gas increases accordingly. Consequently, based on Equations (9)–(12), the heat transfer coefficient between the natural gas and internal surface of the pipeline increases, which, in turn, accelerates the cooling rate of the pipeline HAZ. However, the increasing cooling rate can cause the welding stress to reduce to some degree [32]. When the wall thickness of the pipeline exceeds 12 mm and the natural gas pressure is below 2.5 MPa, the effect of natural gas on the cooling rate of the pipeline HAZ is limited [23]. Additional, considering the effect of the fillet weld, the impact on the cooling rate of the sleeve HAZ will be further attenuated. Moreover, the gas pressure is directly applied on the pipeline wall, placing both the axial and circumferential directions of the pipeline in a tensile state. As a result, as the pressure increases, the tensile stress also rises, further increasing the safety risk of the pipeline.

### 4.4. Effect of the Gas Flow Rate on the Welding Stress

**Figure 19:**
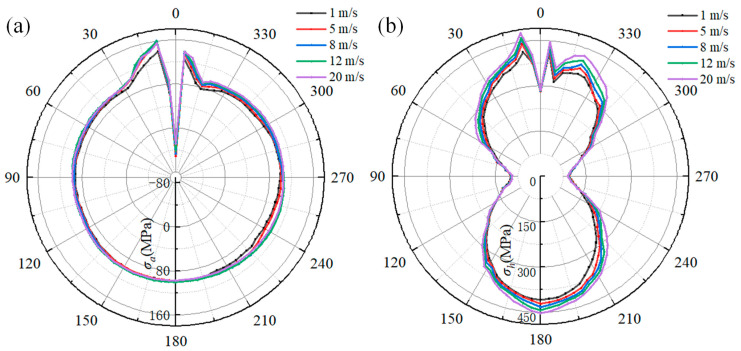
The effect of gas flow rate on the welding stress of the pipeline: (**a**) the axial stress; (**b**) the hoop stress.

The influence of the gas flow rate on the welding stress of the pipeline is shown in Figure 19. Figure 19a depicts the relationship between the gas flow rate and axial stress. When comparing the axial stress distribution along the path, the axial stress at 0° is compressive stress, and the maximum tensile stress is at 9°. As the gas flow rate increases, there are obvious differences in axial stress within a range of 345° to 20° and range of 225° to 260° on the pipeline path. When the flow rate varies from 1 m/s to 20 m/s, the maximum axial stress along the path first rises from 140 MPa to 160 MPa and then decreases to 156 MPa. The axial stress at 240° increases from 78 MPa to 98 MPa. However, the axial stress at 180° remains almost unchanged as the gas flow rate increases. At the 0° location, when the gas flow rate is 5 m/s, the compressive stress is −51 MPa, which is greater than the axial stress for other gas flow rates.

Figure 19b shows the hoop stress along the pipeline path. It is clearly observed that the hoop stress at 90° and 180° is not influenced by the gas flow rate, while the hoop stress at other locations has a positive correlation with the gas flow rate. As the gas flow rate increases from 1 m/s to 20 m/s, the hoop stress at 9°, 180°, and 340° increases from 415 MPa to 479 MPa, 408 MPa to 452 MPa, and 361 MPa to 424 MPa, respectively.

**Figure 20:**
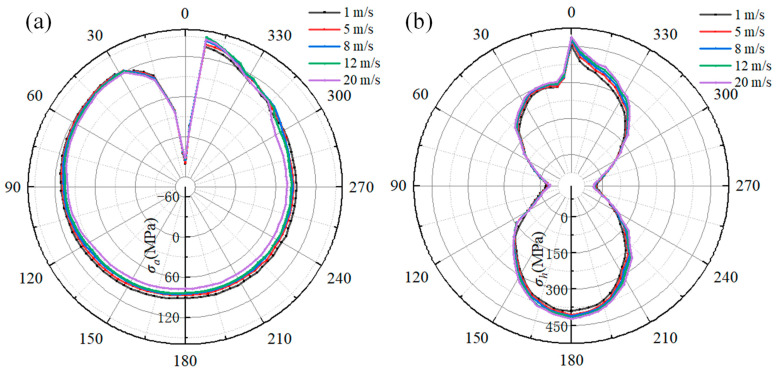
The effect of gas flow rate on the welding stress on sleeve: (**a**) the axial stress; (**b**) the hoop stress.

Figure 20 shows the relationship between the gas flow rate and welding stress along the path of the sleeve. As illustrated in Figure 20a, the axial stress at every location from 0° to 300° decreases as the gas flow rate increases. However, within a range of 300° to 360°, the axial stress at a gas flow rate of 12 m/s is higher than that at other gas flow rates. At the 351° location, the axial stress reaches its maximum value for all gas flow rates. When comparing the axial stress at 351°, it can be observed that the gas flow rate increases from 1 m/s to 20 m/s, and the axial stress first increases from 138 MPa to 151 MPa and then decreases to 147 MPa.

Figure 20b shows the hoop stress along the sleeve path. It can be seen that hoop stress is positively correlated with the gas flow rate. When comparing the hoop stress distribution curves, the most significant difference is at the 345°location due to the influence of the gas flow rate. At 90° and 270°, the compressive hoop stress increases from −25 MPa to −38 MPa as the gas flow rate increases from 1 m/s to 20 m/s. As the gas flow rate increases from 1 m/s to 20 m/s, the peak value of hoop stress at 0° increases from 452 MPa to 488 MPa, the hoop stress at 180° increases from 390 MPa to 423 MPa, and the hoop stress at 345° increases from 330 MPa 384 MPa, respectively.

By comparing the axial and hoop stresses in Figure 19 and Figure 20, it can be found that as the gas flow rate increases from 1 m/s to 20 m/s, the peak axial and hoop stresses along the reference path increase by 11.42% and 15.42%, respectively. Meanwhile, the peak axial and hoop stresses for the sleeve increase by 9.42% and 7.96%, respectively. However, when the gas flow rate is 12 m/s, the peak axial stress along the reference path of both the pipeline and sleeve is larger than that at other gas flow rates. As demonstrated by Equations (9)–(12), the heat transfer coefficient is directly proportional to the natural gas flow rate. Nevertheless, it is important to note that the flow rate has the sole impact on the cooling rate and does not generate a load on the pipeline. Instead, it exerts its influence solely on the microstructure of the HAZ, which, in turn, leads to an alteration in the welding stress [33,34,35]. Despite the fact that the flow rate has an impact on the heat transfer coefficient, once the wall thickness exceeds 12 mm, the influence of the gas flow rate on the cooling rate becomes constrained [23]. Simultaneously, the structure of the fillet weld impairs the heat transfer performance, further reducing the effect of the natural gas flow rate on the cooling rate of the sleeve. As a result, by comparing the welding stress distribution, it can be found that the influence of the gas flow rate on the welding stress of the X80 steel pipeline is more pronounced than that at the sleeve. Notably, the welding stress can be regulated with an appropriate flow rate range.

### 4.5. The Effect of the Preheat Temperature on the Welding Stress

**Figure 21:**
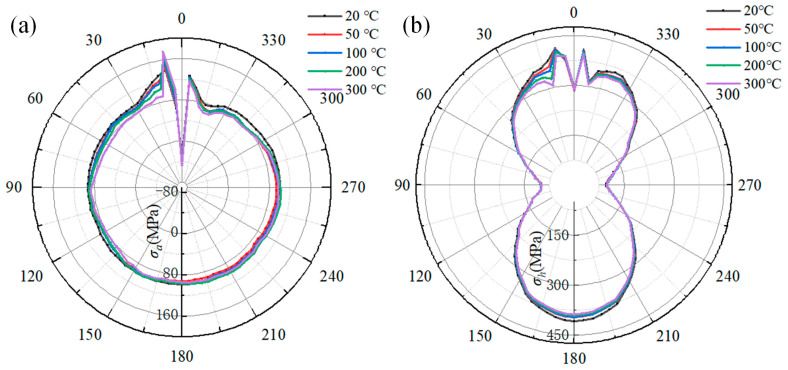
The effect of the preheat temperature on the welding stress on pipeline: (**a**) the axial stress; (**b**) the hoop stress.

Figure 21 illustrates the influence of the preheat temperature on the welding stress of the pipeline. Figure 21a shows the relationship between the axial stress and the preheat temperature. As the preheat temperature increases from 20 °C to 300 °C, the maximum axial stress at 9° rises from 140 MPa to 176 MPa, while the axial stress in a range of 11° to 180° decreases significantly. Moreover, the peak axial stress along the reference path is only increased by about 8 MPa. Especially at the 11° location, the axial stress drop is larger than that at other locations. The axial stress decreases from 131 MPa to 91 MPa as the preheat temperature increases from 20 °C to 300 °C. From the 180° to 300° location, the axial stress at a preheat temperature of 50 °C is lower than that at other preheat temperatures. When comparing the axial stress in a range of 300° to 360°, the axial stress is negatively correlated with the preheat temperature again. At 356°, the axial stress decreases from 126 MPa to 117 MPa.

Figure 21b shows the effect of the preheat temperature on the hoop stress of the pipeline. It can be seen that the increasing preheat temperature has no obvious effect on the hoop stress in a range of 45° to 150° and 210° to 315°. At a preheating temperature of 50 °C, the peak hoop stress along the reference path is almost equivalent to that corresponding to the preheating temperature of 20 °C. However, there is a significant difference in hoop stress at the 12° location. The hoop stress decreases from 382 MPa to 307 MPa as the preheat temperature increases from 20 °C to 300 °C. At the 9° location, the peak hoop stress along the path decreases from 415 MPa to 393 MPa. Therefore, preheating can effectively reduce the axial stress and hoop stress on the pipeline, although the peak axial stress at 9° increases under the influence of preheat temperature.

**Figure 22:**
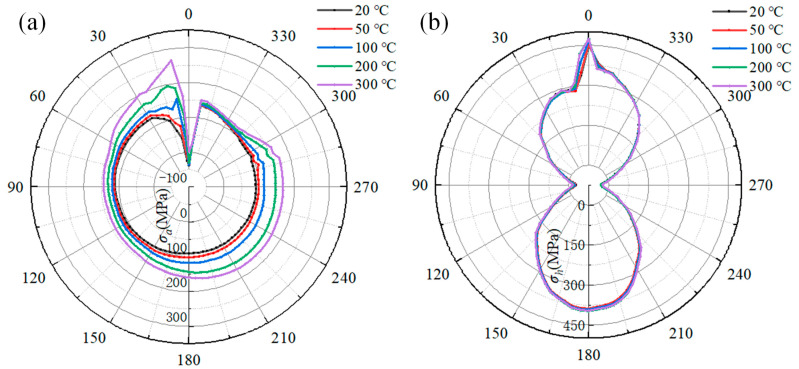
The influence of the preheat temperature on the welding stress on the sleeve: (**a**) the axial stress; (**b**) the hoop stress.

Figure 22 presents the welding stress on the sleeve at different preheat temperatures. In Figure 22a, the axial stress at 0° becomes tensile when the preheat temperature exceeds 300 °C. As the preheat temperature increases from 20 °C to 300 °C, there are obvious changes in the stress curves, except for the range of 330° to 360°. The axial stress curves expand, and the stress at each location increases. It is obviously found that the hoop stress at 9° is most significantly affected by the preheat temperature. It increases from 41 MPa for 20 °C to 267 MPa at 300 °C, an increase of 226 MPa. However, when the preheating temperature reaches 50 °C, the peak hoop stress along the reference path is roughly 9 MPa greater than that at a preheating temperature of 20 °C.

As depicted in Figure 22b, there is no distinct variation in the hoop stress at each angle within a range of 9° to 360°. However, the hoop stress in a range of 0° to 9° increases as the preheat temperature rises. At the 0° location, which represents the peak value of hoop stress on the reference path, the hoop stress increases from 452 MPa to 474 MPa, with the preheat temperature increasing from 20 °C to 300 °C. Moreover, when the preheating temperature is 50 °C, the peak hoop stress along the reference path is 4 MPa higher than that at 20 °C.

A comparative analysis of how the preheating temperature impacts the welding stress in the pipeline and sleeve yielded the following findings. When the preheating temperature reaches 50 °C, the axial and hoop peak stresses along reference path of the pipeline experience increases of 5.71% and 0.1%, respectively, as compared to the peak stress at 20 °C. Similarly, for the sleeve, the axial and hoop peak stresses along its reference path rise by 6.85% and 0.9%, respectively, relative to the values at 20 °C. When then preheating temperature climbs to 300 °C, the pipeline’s axial peak stress along the reference path surges by 25.27%, yet its hoop peak stress drops by 5.3% compared to the 20 °C level. In contrast, for the sleeve, the axial and hoop peak stresses along the reference path soar by 115.3% and 4.87%, respectively, in comparison to the peak stress at 20 °C.

It is found that the axial stress is affected more significantly than hoop stress. Specifically, the axial stress on the sleeve is prominent. When the pipeline and sleeve are preheated, the cooling rate of the pipeline is faster than that of the sleeve. This is because the natural gas within the pipeline can carry away a large amount of heat. Moreover, during the filling pass deposition, the majority of the welding-induced heat is transferred to the sleeve. Notably, as indicated in reference [36], the preheat temperature can cause changes in the microstructure composition, which, in turn, influence the welding stress. Additionally, the fillet welds at both ends of the sleeve restrain the axial expansion of the sleeve, further contributing to the complex stress state in the sleeve. Therefore, by controlling the preheating temperature within a range of 50 °C to 100 °C, the welding stress in both pipelines and sleeves can be effectively managed, ensuring the quality and integrity of the welded structures.

## 5. Conclusions

A 3D finite element simulation of in-service welding on an X80 streel pipeline was established, and the parameters of the heat source model and welding stress were verified. The findings led to the following conclusions:

1. The distribution patterns of axial and hoop stresses are remarkably consistent at the HAZ location on both the inner and outer walls of the pipeline and sleeve. In the HAZ location, the outer walls of the pipelines and sleeve are predominantly under compressive stress, while the inner walls are mainly subjected to tensile stress.
2. The maximum axial stress at the fillet weld is predominantly located on the side of the sleeve’s HAZ, and the maximum hoop stress is principally concentrated at the connection point between the fillet weld and the sleeve. When compared with the axial stress, the hoop stress is significantly greater and exceeds the yield strength of the X80 pipeline steel. This phenomenon serves as the primary contributor to the cracking of in-service welding fillet welds.
3. The peak values of both the axial and hoop stresses are predominantly concentrated in the start–end region. At the start–end region of the weld, a significant abrupt change takes place in the axial and hoop stresses of the pipeline and sleeve. When the start–end region is excluded, the axial stress distributed in the circumferential direction remains relatively constant. The hoop stress values demonstrate an approximately symmetric distribution, with relatively higher values at 0° and 180° and relatively lower values at 90° and 270°.
4. During the in-service welding of X80 natural gas pipelines, as the natural gas pressure and flow rate increase, the axial stress on the pipeline and sleeve first shows an upward trend and then a downward one. In contrast, the hoop stress exhibits a continuously increasing trend. Specifically, when the natural gas pressure increases from 0.5 MPa to 2.5 MPa, the hoop stress of the pipeline and sleeve increases by 3.18% and 1.17%, respectively. Similarly, when the natural gas flow rate surges from 1 m/s to 20 m/s, the hoop stress of the pipeline and sleeve rises by 15.42% and 7.96%, respectively. To effectively mitigate the risk of welding-induced stress caused by natural-gas-related factors during pipeline operation, it is recommended to maintain the natural gas pressure at 1 MPa and the flow rate below 12 m/s.
5. When the preheating temperature is elevated from 20 °C to 300 °C, the hoop stress of the pipeline and sleeve experiences changes of −5.3% and 4.87%, respectively. Meanwhile, the axial welding stress increases by 25.27% and 115.3%, respectively. To effectively prevent the degradation of welding stress, it is advisable to control the preheating temperature at approximately 50 °C during the in-service welding of an X80 steel pipeline.

## Figures and Tables

**Table 1:** Thermal physical parameters of X80 pipeline steel.

Temperature (°C)	Density (kg·m^−3^)	Thermal Conductivity (W·m^−1^·°C^−1^)	Specific Heat (J·kg^−1^·°C^−1^)	Coefficient of Linear Expansion (10^−5^·°C^−1^)	Poisson’s Ratio (-)	Yield Strength (MPa)	Elastic Modulus (GPa)
20	7810	54.42	423	1.10	0.29	590	210
100	7790	54.01	473	1.15	0.29		207
200	7770	52.75	536	1.22	0.29	490	204
400	7720	47.71	662	1.35	0.29	385	187
500					0.29	113	142
600					0.29	108	138
700					0.29	103	120
800	7610	27.55	914		0.29		
1200	7500	40.00	1160		0.29		

**Table 2:** Thermal physical parameters of water.

Temperature (°C)	Density (kg·m^−3^)	Thermal Conductivity (W·m^−1^·°C^−1^)	Specific Heat (J·kg^−1^·°C^−1^)	*Pr* (-)	Dynamic Viscosity (×10^−6^ Pa·s)
0	999.9	0.551	4212	13.67	1788
10	999.7	0.574	4191	9.52	1306
20	998.2	0.599	4183	7.02	1004
30	995.7	0.618	4174	5.42	801.5
40	992.2	0.635	4174	4.31	653.3
50	988.1	0.648	4174	3.54	549.4
60	983.1	0.659	4179	2.99	469.9
70	977.8	0.668	4187	2.55	406.1

**Table 3:** Welding parameters.

Welding Pass	Root Pass	Filling Pass	Overlaying Pass
Welding method	SMAW	FMAW-G	FMAW-G
Electrode material	E8018-C3	E91T1-GM	E91T1-GM
Electrode diameter (mm)	Φ3.2	Φ1.2	Φ1.2
polarity	DCEP	DCEP	DCEP
Voltage (V)	22–28	20–26	19–26
Current (A)	100–130	180–240	130–180
Welding speed (cm/min)	6–15	15–25	18–25
Gas flow (L/min)		20–30	20–30
Heat input (kJ/mm)	0.85~1.8	1.6~3.2	0.8~1.9

**Table 4:** The physical property parameters of methane in the pipe.

Temperature (°C)	Pressure (MPa)	Density (kg/m^3^)	Specific Heat (kJ/(kg·°C))	Thermal Conductivity (W/(m·°C))	Dynamic Viscosity (×10^−6^ Pa·s)	*Pr* (-)
0	2	14.8	2.335	0.0321	10.56	0.768
	6	49.2	2.782	0.0366	11.67	0.887
	8	68.9	3.068	0.0399	21.53	0.964
20	0.5	2.54	2.301	0.0355	10.65	0.69
	1	4.75	2.318	0.0357	10.74	0.697
	2	13.7	2.345	0.036	11.20	0.759
	6	44.1	2.674	0.0384	12.16	0.846
	8	60.8	2.867	0.0410	12.85	0.898

**Table 5:** The parameters of DEHSM.

Welding Pass No.	*a*_r_	*a*_f_	*b*	*c*
1	6.7	13.3	11	4
2	6.7	13.3	12	4
3	7	14	15	4
4	7	14	16	4
5	6.7	13.3	10	4
6	8	16	16	5
7	8	16	16	5
8	8	16	16	5
9	8	16	16	5
10	8	16	16	5
11	8	16	16	5

**Table 6:** The test hardness of reference point.

Point	1	2	3	4	5	6	7	8	9	10	11	12	13
Hv_10_	215	203	245	194	228	222	213	225	210	221	232	210	213

## Data Availability

The original contributions presented in this study are included in the article; further inquiries can be directed to the corresponding author.
